# An anti-CD47 antibody binds to a distinct epitope in a novel metal ion-dependent manner to minimize cross-linking of red blood cells

**DOI:** 10.1016/j.jbc.2025.110420

**Published:** 2025-06-25

**Authors:** Xiao Lu, Ziyue Chen, Chunyan Yi, Zhiyang Ling, Jing Ye, Kaijian Chen, Yao Cong, Wangmo Sonam, Shipeng Cheng, Ran Wang, Danyan Zhang, Jiefang Xu, Jichao Yang, Liyan Ma, Qing Duan, Xiaoyu Sun, Jianping Ding, Bing Sun

**Affiliations:** 1 Key Laboratory of Multi-Cell Systems, Shanghai Institute of Biochemistry and Cell Biology, Center for Excellence in Molecular Cell Science, University of Chinese Academy of Sciences, Chinese Academy of Sciences, Shanghai, China; 2 Key Laboratory of RNA Innovation, Science and Engineering, Shanghai Institute of Biochemistry and Cell Biology, Center for Excellence in Molecular Cell Science, University of Chinese Academy of Sciences, Chinese Academy of Sciences, Shanghai, China; 3 School of Life Science and Technology, ShanghaiTech University, Shanghai, China; 4 Division of Life Sciences and Medicine, University of Science and Technology of China, Hefei, China; 5 Research & New Technology Department, BioDlink Biopharm Co., Ltd, Suzhou, China; 6 Shanghai Institute of Infectious Disease and Biosecurity, Shanghai Medical College, Fudan University, Shanghai, China

**Keywords:** bivalent metal ion, CD47, cell cross-linking, crystallography, non-RBC hemagglutination, rabbit monoclonal antibody

## Abstract

Cluster of differentiation 47 (CD47) is a widely expressed transmembrane protein that plays a crucial role in immune self-recognition. Cancer cells upregulate CD47 expression to promote immune escape through activating the “don’t eat me” signal *via* interactions with signal regulatory protein α (SIRPα) on macrophages. The effectiveness of anti-CD47 antibodies has been demonstrated in multiple tumor models. However, since CD47 is also expressed in human red blood cells (RBCs) and platelets, the clinical application of anti-CD47 antibodies requires careful consideration of blood toxicity. One major obstacle to the clinical application of CD47 antibodies is the hemagglutination caused by RBCs cross-linking. In this study, we generated Hu1C8, a humanized anti-CD47 monoclonal antibody that demonstrated increased selectivity for binding to CD47 on cancer cells and lacked hemagglutination activity. Epitope mapping and the crystal structure of the Hu1C8 Fab-CD47 extracellular domain (ECD) complex revealed that Hu1C8 binds to a distinct epitope of CD47 in a Ca^2+^-dependent manner. The unique recognition and binding mode allowed Hu1C8 to bind CD47 on RBCs with reduced hemagglutination activity while still maintaining effective antitumor activity. These findings demonstrate a feasible strategy for developing CD47 antibodies with high antitumor activity but low RBC hemagglutination activity. Our study elucidates how epitope-specific antibody influences antibody-induced cell cross-linking, offering innovative strategies for antibody design to either leverage or avoid cell cross-linking effects.

CD47, which is formerly known as integrin-associated protein (IAP) (1), is a cell surface glycoprotein that is ubiquitously expressed on human cells (2, 3). It has been implicated in the regulation of multiple cellular processes involved in immune responses, such as the inhibition of innate immune cells and cell migration (2). SIRPα is the inhibitory receptor of CD47 expressed on myeloid cells (4, 5), including macrophages, dendritic cells (DCs) and neutrophils, and plays a crucial role in the regulation of innate immune responses and phagocytosis (3, 6). The CD47/SIRPα interaction provides a “don’t eat me” signal that inhibits phagocytosis. CD47 has been identified as a tumor antigen that is expressed by multiple human tumor types, including leukemia (7), lymphoma (8), myeloma (9), and certain solid tumors (10, 11, 12). Many studies have suggested that the CD47/SIRPα axis acts as an innate immune checkpoint in macrophages (13, 14). Cancer cells overexpress CD47 to evade immune surveillance; thus, CD47 can be a target for immunotherapy (15). Blocking the CD47/SIRPα axis has been shown to enhance phagocytosis of tumor cells *in vitro* and promote macrophage-mediated tumor elimination in multiple *in vivo* tumor models (7, 16, 17). These findings suggest that targeting this axis may be a promising approach for cancer immunotherapy. However, CD47 is also expressed on normal cells, particularly on RBCs and platelets; thus, some anti-CD47 antibody therapies have shown significant side effects, including hemagglutination and the phagocytosis of RBCs (18, 19). The on-target off-tumor effects of anti-CD47 antibody therapeutics significantly impact the intended functions of the antibodies. Therefore, the development of anti-CD47 antibodies with minimized RBC binding and toxicity, while retaining potent activity against tumor cells, represents a promising approach for advancing CD47-targeted cancer immunotherapy.

In this study, we generated 1C8, an anti-CD47 antibody with minimal hemagglutination activity, using rabbit single B-cell screening technology. Following humanization, the resulting antibody was named Hu1C8. Functional studies showed that Hu1C8 exhibited similar activity to other anti-CD47 antibodies in the clinical stage by promoting tumor cell phagocytosis *in vitro* and exerting antitumor effects *in vivo*. In addition, Hu1C8 exhibited a reduced risk of RBC toxicity due to its low hemagglutination activity. Structural analysis revealed that Hu1C8 binds to the proximal membrane region of CD47 with a unique binding angle, effectively preventing the antibody arms from simultaneously engaging CD47 on adjacent cells, thus avoiding cell cross-linking. Notably, the binding of Hu1C8 to CD47 depends on Ca^2+^, which has been rarely reported before.

In conclusion, we have demonstrated the therapeutic potential of the humanized rabbit-derived monoclonal anti-CD47 antibody Hu1C8. Crystal structure analysis revealed the Ca^2+^-dependent recognition pattern of Hu1C8. This pattern involves a unique binding angle of one Hu1C8 Fab arm to CD47, which restricts the accessibility of the other arm of the antibody, thus inhibiting antibody-induced RBC cross-linking. In addition, it provides new antibody design strategies for utilizing or circumventing cell cross-linking.

## Results

### Generation of rabbit-derived monoclonal antibodies against human CD47

**Figure 1:**
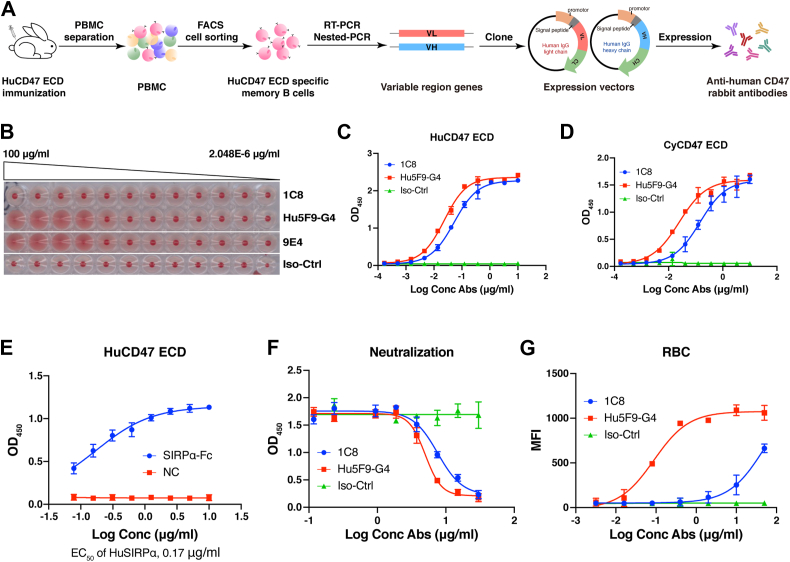
**Flowchart of rabbit single-cell memory B-cell technology and the characterization of 1C8.***A*, flowchart of rabbit single-cell memory B-cell technology. *B*, hemagglutination activity of 1C8. Other CD47 antibodies, Hu5F9 and 9E4 (Reference US 2014/0,140,989 A1) associated with hemagglutination issue, were used as controls. *C*, binding activity of 1C8 to the HuCD47 ECD. *D*, binding activity of 1C8 to the CyCD47 ECD. *E*, binding activity of SIRPα-Fc to the HuCD47 ECD. NC, Unrelated proteins that do not bind to HuCD47 ECD. *F*, neutralizing activity of 1C8 towards the HuCD47 ECD. *G*, binding activity of 1C8 to CD47 on human RBCs. Iso-Ctrl, isotype control antibody. The results in (*C*, *D*, *E*, *F*, *G*) are presented as the means ± SD. Data are representative of two or three independent experiments.

The ECD of human CD47 (HuCD47 ECD) was used to immunize rabbits and produce rabbit CD47 monoclonal antibodies (Fig. 1*A*). We first measured the antigen binding abilities of the plasma derived from immunized rabbits. Initial evaluation of immunized antisera by enzyme-linked immunosorbent assay (ELISA) revealed a >10,000-fold increase in antigen-binding activity compared to pre-immunized antisera (data not shown). For antibody isolation, we performed single-cell polymerase chain reaction (PCR) experiments to isolate rabbit monoclonal antibodies from immunized rabbits using the biotinylated HuCD47 ECD as the capture reagent. Flow cytometry sorting (FACS) was employed to isolate single CD4^-^CD8^-^IgM^-^IgG^+^HuCD47 ECD^+^ memory B cells. The variable regions of selected antibodies generated directly from single memory B cells were reconstructed with the constant region of human IgG4PE (IgG4-S228P/L235E) to facilitate humanization, followed by assessment of the binding and neutralizing activities with CD47 (data not shown).

Anti-CD47 antibodies may cause homotypic clustering of RBCs (hemagglutination), which is one of the reasons for RBC toxicity. To identify antibodies with low RBC toxicity, we performed a human RBC agglutination experiment to select CD47 antibodies that did not induce hemagglutination. Human RBCs were diluted to 2% in phosphate buffer saline (PBS) and incubated with different concentrations of CD47 antibodies at room temperature for 1 to 2 h. Hemagglutination is characterized by the presence of dispersed RBCs, while RBCs that did not undergo hemagglutination precipitate and form red dots at the bottom of the plates. We screened nearly 20 CD47-specific antibodies; only 1C8 showed no hemagglutination (Fig. 1*B*) and was selected as a candidate for humanization and further in-depth characterization.

ELISA and FACS-based experiments were performed to evaluate 1C8 *in vitro*. A humanized mouse anti-human CD47 antibody, Hu5F9-G4, which is in the clinical trial stage (20), and an isotype antibody that binds to hepatitis C virus (21) were used as positive and negative controls, respectively. The antigen-binding affinity and neutralizing capacity of 1C8 were quantified by ELISA, demonstrating no significant difference from Hu5F9-G4 in both assays. (Fig. 1, *C* and *F*). Prior to neutralizing efficacy evaluation, we pre-tested the activity of SIRPα to ensure the reliability of the experiment (Fig. 1*E*). In addition, 1C8 cross-reacted with the ECD of cynomolgus CD47 (CyCD47 ECD) (Fig. 1*D*). Cell-based binding was examined with human RBCs. The results showed that the binding activity of 1C8 to RBCs was significantly lower than that of Hu5F9-G4 (Fig. 1*G*). In conclusion, the 1C8 antibody showed robust binding activity to human and cynomolgus CD47 but weak binding to RBCs. These properties make it an ideal candidate for a therapeutic CD47 antibody.

### Humanization of 1C8

**Figure 2:**
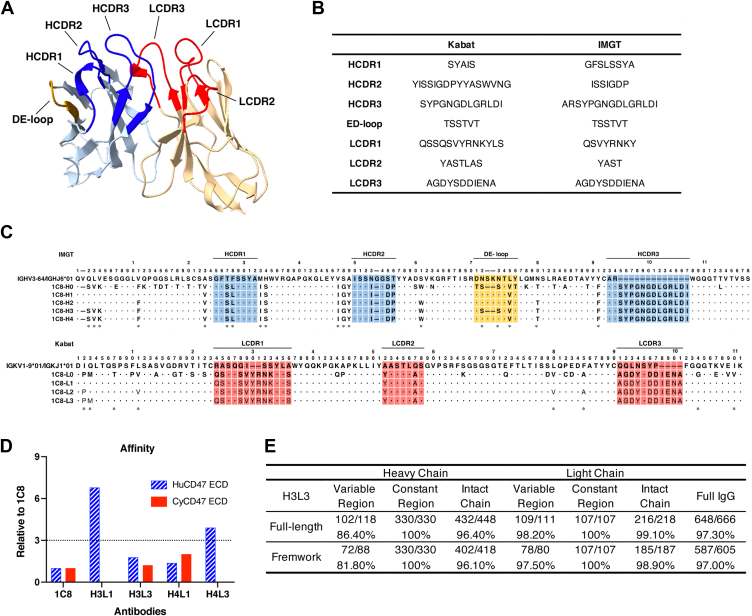
**Humanization of 1C8.***A*, the structural model of 1C8. The CDR regions of 1C8 VH and Vκ are coloured blue and red, respectively. *B*, the sequences of the 1C8 CDR regions were obtained from the Kabat and IMGT databases. *C*, the amino acid sequence alignments of VH and Vκ of the humanized variant sequences with human germline sequences. *D*, the relative binding affinity of the humanizing antibodies for the original antibody 1C8. *E*, the degree of humanization of H3L3.

To humanize 1C8, a 3D structure model was first built using combinations of known structures (PDB codes: 6BA5 (22), 5I8K, 5V6M (23), 6I9I (24)) that share more than 80% sequence identity with 1C8 (Fig. 2*A*). The online tools Kabat and IMGT were used to identify the complementarity-determining region (CDR) residues. Combined with the structure model information, the heavy chain CDRs (HCDRs) were referenced to IMGT, and the light chain CDRs (LCDRs) were referenced to Kabat (Fig. 2, *A* and *B*). The 1C8 variable genes (VH and Vκ) were subsequently queried using Ig-BLAST against the human germline VH and Vκ databases to identify appropriate human antibody framework regions (FRs) that were suitable for use as templates in CDR grafting. IGHV3/IGHJ6 and IGKV1/IGKJ1 were ultimately identified as templates for 1C8 humanization (Fig. 2*C*). Next, the key residues in the FRs that may be involved in CDR contacts and interchain contacts and were buried inside based on analysis of the structure model, were marked with an asterisk (∗) (Fig. 2*C*). After CDR grafting, these residues were considered to be back-mutated. Finally, we designed a series of humanized variants (Fig. 2*C*).

The humanized heavy chains and light chains were recombined with parental light chains (1C8-L0) and heavy chains (1C8-H0) into new antibodies, and the reactivity of the humanized antibody variants was subsequently examined. The ELISA-binding results indicated that H1L0 and H2L0 lost all or part of their binding activity (Fig. S1, *A* and *B*). After removing 1C8-H1 and 1C8-H2, the remaining humanized heavy and light chains could recombine to form new antibodies. The binding and neutralizing activity assay results showed that these antibodies had similar EC_50_ values (the concentration for 50% of the maximal effect); however, the IC_50_ values (the half maximal inhibitory concentration) of H3L2 and H4L2 were slightly higher than that of 1C8 (Fig. S1, *C*–*E*). We further assessed the binding kinetics of the antibodies to HuCD47 ECD and CyCD47 ECD using an Octet RED96 instrument. The results revealed that both H3L1 and H4L3 had a 3-fold lower binding affinity to the HuCD47 ECD than the parental antibody 1C8, which did not meet the humanization standard, and the binding affinity (*K*_d_) values of H3L3 and H4L3 for the HuCD47 ECD and CyCD47 ECD met the humanization standard (Figs. 2*D*; S1, *F*–*N*). H3L3 was closer to 1C8 according to the *K*_on_ (association rate constant) and *K*_off_ (dissociation rate constant) parameters and was selected as the final humanized antibody (Hu1C8) (Figs. 2*D*; S1*N*). After engineering, the intact chains of Hu1C8 were 96.1% and 98.9% identical to the human heavy chain and light chain, respectively (Fig. 2*E*).

### Functional characterization of the humanized anti-CD47 antibody Hu1C8 *in vitro*

**Figure 3:**
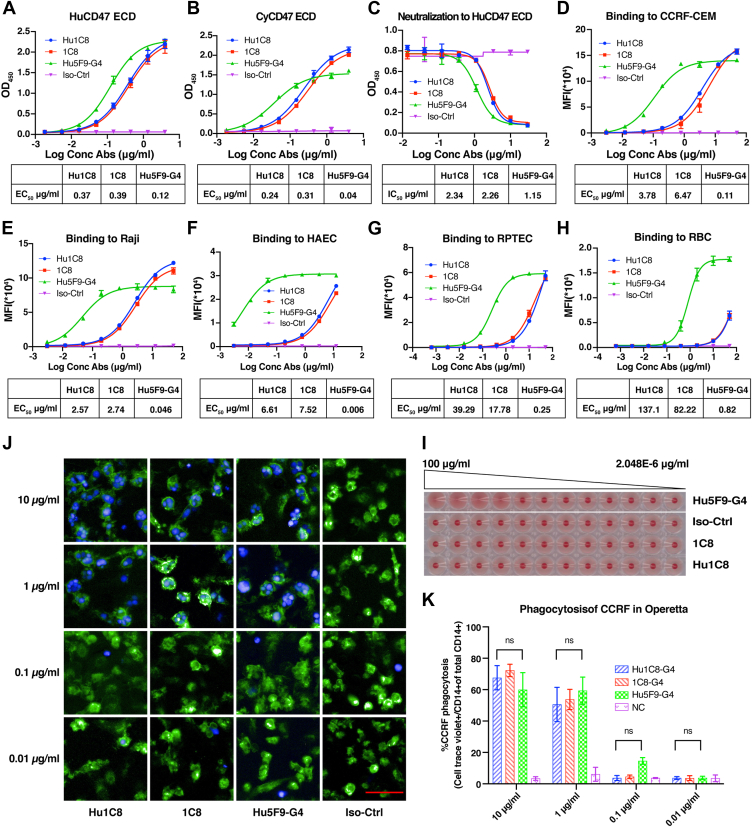
**Functional characterization of the humanized anti-CD47 antibody Hu1C8.***A* and *B*, binding activities of Hu1C8 to the HuCD47 ECD (*A*) and CyCD47 ECD (*B*). *C*, neutralization activity of Hu1C8 towards the HuCD47 ECD. *D*–*H*, binding activities of Hu1C8 to CD47 on CCRF-CEM cells (*D*), Raji cells (*E*), HAECs (*F*), RPTECs (*G*), and human RBCs (*H*). *I*, hemagglutination activity of Hu1C8. *J*, images of macrophage-mediated phagocytosis (20∗). *Green* fluorescence indicates macrophages, *blue* fluorescence indicates tumor cells, and *blue* fluorescence surrounded by *green* fluorescence represents macrophages that engulfed tumor cells. Scale bars represent 100 μm. *K*, statistical analysis of the proportion of phagocytosis. The results in (*A*–*H*, *K*) are presented as the means ± SD. Statistical analyses in (*K*) were performed with two-way ANOVA. “ns” indicates no significant difference. Data are representative of two or three independent experiments.

The functional activity of Hu1C8 *in vitro* was characterized in the same manner as 1C8. The antigen binding and neutralization activities of Hu1C8 were examined by ELISA (Fig. 3, *A*–*C*). The binding affinities of Hu1C8 and 1C8 were evaluated by FACS using two lymphoma models: CCRF-CEM cells (human T lymphoblasts derived from a 4-year-old female T-ALL patient) and Raji cells (a Burkitt’s lymphoma B-cell line). As shown in Figure 3, *D* and *E*, Hu1C8 exhibited similar binding activity to 1C8.

To compare the binding of Hu1C8 to normal and tumor cells, we also used a flow cytometry-based binding assay using human aortic endothelial cells (HAECs), human renal cortex proximal tubule epithelial cells (RPTECs), and human RBCs (Fig. 3, *F*–*H*). The relative EC_50_ of Hu1C8 for normal cells *versus* tumor cells was significantly higher than that of Hu5F9-G4 (Figs. 3, *D*–*H*; S2*A*). The results indicate that Hu1C8 has lower binding activities to normal cells, especially RBCs, than to tumor cells. The selective binding is expected to have advantages over antibodies that bind with similar affinity to both normal and tumor cells.

Like 1C8, Hu1C8 did not cause any hemagglutination. In contrast, Hu5F9-G4 caused significant hemagglutination, as previously reported (Figs. 3*I*; S2, *B*–*D*).

We next sought to determine the ability of Hu1C8 to induce macrophage-mediated phagocytosis of tumor cells. Antibody-dependent cellular phagocytosis (ADCP) analysis was performed using CCRF-CEM cells as the targets and macrophages derived from healthy human peripheral blood mononuclear cells (PBMCs) as the effector cells. The results showed that 1C8 and its humanized variant Hu1C8 induced dose-dependent increases in the phagocytosis of tumor cells. At concentrations of 0.1 μg/ml, 1 μg/ml, and 10 μg/ml, Hu1C8 and Hu5F9-G4 induced macrophage-mediated phagocytosis of CCRF-CEM cells to similar extents (Fig. 3, *J* and *K*). These data demonstrate that Hu1C8 induces macrophage-mediated phagocytosis of CD47-positive tumor cells. Taken together, these results show that Hu1C8 retains all the biological activities of its parental antibody *in vitro*.

### Hu1C8 effectively inhibits tumor growth in NSG mice

**Figure 4:**
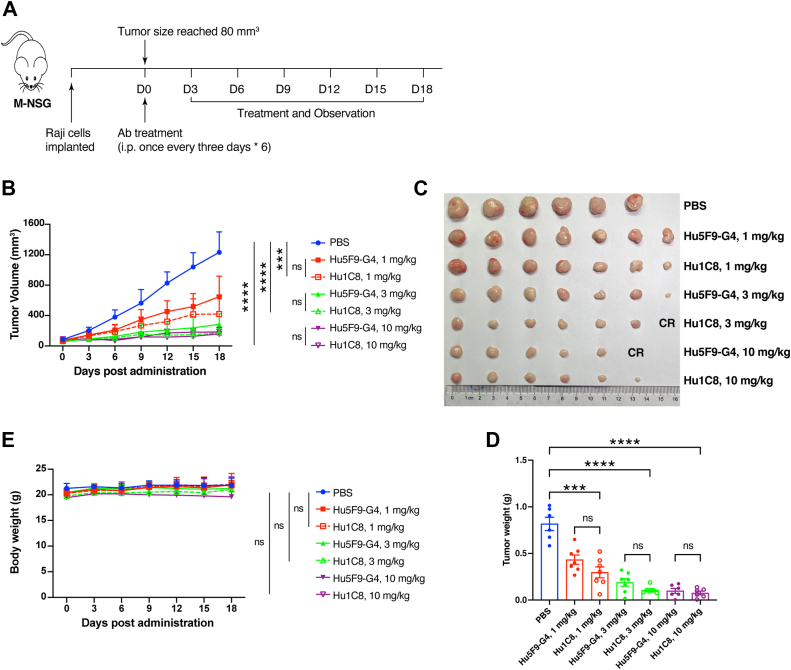
**Hu1C8 inhibited tumor growth *in vivo*.***A*, schematic diagram showing tumor inoculation and antibody treatment of the mice. *B*, tumor volume under treatment with varying antibody doses. *C*, images of tumor tissues. CR, Complete Response. *D*, tumor weight. *E*, the curves of body weight. The results are presented as the means ± SD, (n = 6–7 mice per group). Statistical analyses in (*B*, *D*, *E*) were performed with an unpaired *t* test. ∗*p* < 0.05, ∗∗*p* < 0.01, ∗∗∗*p* < 0.001, ∗∗∗∗*p* < 0.0001, “ns” indicates no significant difference. Data are representative of two or three independent experiments.

To evaluate the *in vivo* antitumor activity of Hu1C8, the Raji lymphoma model was treated with antibodies. Raji cells were implanted subcutaneously into M-NSG mice (NOD.Cg-*Prkdc*^*scid*^*Il2rg*^*em1Smoc*^; Shanghai model organisms). After the tumor size reached 80 mm^3^, the mice were randomly divided into seven groups and received antibody treatment. The antibodies were intraperitoneally injected once every 3 days for six continuous doses. Body weight and the width and length of the tumors were measured every 3 days (Fig. 4*A*). The data demonstrated that all the Hu1C8 treatment groups exhibited significantly reduced Raji tumor volume and tumor weight compared to the vehicle group (Fig. 4, *B*–*D*). Dose–response analyses revealed similar antitumor efficacy between Hu1C8 and Hu5F9-G4 (Figs. 4, *B*–*D*; S3, *A*–*G*). Moreover, no abnormal body weight changes were observed relative to vehicle controls (Fig. 4*E*). These results demonstrate that Hu1C8 can inhibit tumor growth.

### Crystal structure of the Hu1C8 Fab-CD47 ECD complex reveals the molecular mechanism of Hu1C8 bearing low hemagglutination and high antitumor activity

**Figure 5:**
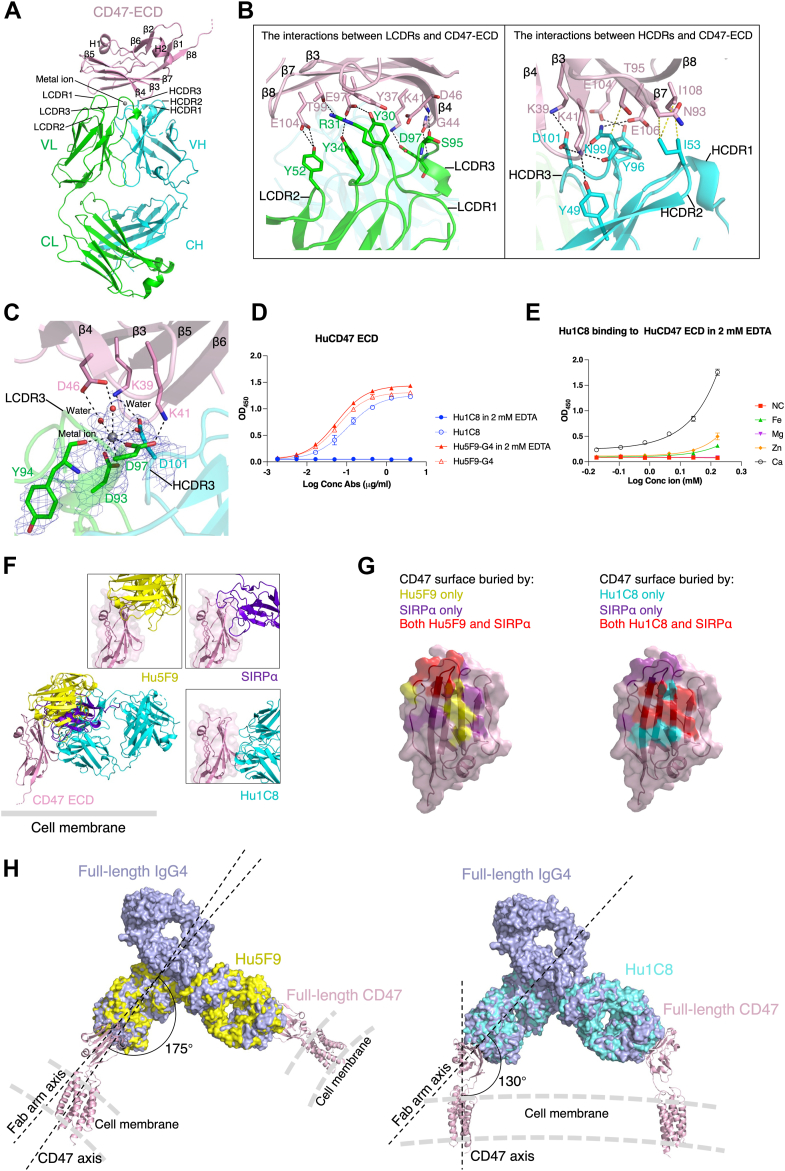
**Crystal structure of the Hu1C8 Fab in complex with the CD47 ECD.***A*, overall structure of the Hu1C8 Fab-CD47 ECD complex shown in ribbon diagram. The heavy and light chains of the Hu1C8 Fab are shown in *cyan* and *green*, respectively. The CD47 ECD is shown in *pink*. *B*, interactions of the CD47 ECD with the light chain (*left*) and heavy chain (*right*) of Hu1C8 Fab. The salt bridges and hydrogen bonds are shown with *black dashed lines*, and the hydrophobic interactions are shown with *yellow dashed lines*. *C*, detailed structure of the CDR region of Hu1C8 involved in the metal ion binding. The 2*F*_*O*_*-F*_*C*_ electron density (contoured at 1.0 σ level) for the metal ion and the interacting residues is shown with a slate-blue mesh. *D*, binding activity of Hu1C8 to HuCD47 ECD in the presence of 2 mM EDTA. The results are presented as the means ± SD. *E*, the addition of metal ions restored the binding activity of Hu1C8 to HuCD47 ECD in the presence of 2 mM EDTA. The results are presented as the means ± SD of two independent experiments. *F*, structural comparison of the Hu1C8 Fab-CD47 ECD complex with the SIRPα-CD47 ECD complex (PDB code: 2JJS) (26) and the Hu5F9 diabody-CD47 ECD complex (PDB code: 5IWL) (25). The superposition of the three complexes was based on the CD47 ECD. The Hu5F9 diabody is a fusion protein of the heavy and light variable domains of Hu5F9. For clarity, only one domain from each diabody is shown. *G*, the CD47-binding interface showing residues interacting with only SIRPα (*purple*), only Hu5F9 (*yellow*), only Hu1C8 (*cyan*), or both ligands (*red*). *H*, the structures of the Hu1C8 Fab-CD47 ECD complex and the Hu5F9 diabody-CD47 ECD complex were superimposed on the structures of whole IgG4 antibody (PDB code: 5DK3) (45) and full-length CD47 (PDB code: 7MYZ) (46), showing that Hu1C8 and Hu5F9 bind to CD47 in different binding orientations, thus yielding different RBC toxicities.

To understand the molecular mechanism by which Hu1C8 induces low hemagglutination but exhibits high antitumor activity, we determined the crystal structure of the Hu1C8 Fab fragment in complex with the CD47 ECD (amino acids Gln1-Pro121 of HuCD47 with C15A mutation) at a resolution of 2.5 Å. Structural analysis of the Hu1C8 Fab-CD47 ECD complex showed that the Hu1C8 Fab recognized a unique conformational epitope on the CD47 ECD (Fig. 5*A*). The L1, L2, L3, H2, and H3 CDR loops of Hu1C8 form a large, shallow pocket to accommodate the CD47 epitope, while the H1 CDR loop does not participate in this interaction (Fig. 5*B*). The conformational epitope of CD47 consists mainly of two structural segments from the β-sheet of the ECD, namely, residues 37 to 46 (β3, β4) and 97 to 108 (β7, β8) (Fig. 5*B*). The interacting interfaces between Hu1C8 and CD47 are dominated by hydrophilic interactions and bury approximately 756 Å^2^ of the solvent-accessible surface area of Hu1C8 and CD47. In particular, eight residues of CD47 (Tyr37, Lys39, Lys41, Asp46, Glu97, Thr99, Glu104, and Glu106) form a number of salt bridges and hydrogen bonds with several residues of LCDR1 (Tyr30 and Arg31), LCDR2 (Tyr52), LCDR3 (Asp97), and HCDR3 (Tyr96, Asn99, and Asp101) (Fig. 5*B*). The side chain of Tyr37 is also stabilized by a cation-π interaction with Arg31 of LCDR1. In addition to the electrostatic and hydrogen-bonding interactions, a small patch of hydrophobic interactions is also observed, including Asn93 and Thr95 of CD47-β7, Ile105 of CD47-β8, Ile53 of HCDR2, and Asn99 of HCDR3 (Fig. 5*B*). To support that this conformational epitope does mediate Hu1C8-CD47 ECD complex binding, we performed alanine-scanning mutagenesis of CD47 ECD. ELISA-based binding analysis showed that alanine substitution of Tyr37, Lys39, Lys41, Asp46, and Glu106 markedly reduced the binding of Hu1C8 to CD47 (Fig. S4). We also found that the N93A, T95A, and I108A mutations of CD47 had insignificant effects on the binding (Fig. S4). These results indicate that the hydrophilic interactions dominate the binding affinity of the Hu1C8-CD47 ECD interaction.

Intriguingly, we found a metal ion bound at the CDR region of Hu1C8, which is coordinated by the side-chain Oδ1 and Oδ2 of Asp93, the main-chain carbonyl of Tyr94, the side-chain Oδ1 of Asp97 on LCDR3, the side-chain Oδ1 of Asp101 on HCDR3, and two water molecules with a pentagonal bipyramid coordination geometry (Fig. 5*C*). As the side chains of Asp97 on LCDR3 and Asp101 on HCDR3 and the two water molecules not only serve as ligands for the metal ion but also form hydrophilic interactions with the epitope residues Lys39, Lys41, and Asp46 of CD47, we speculated that the binding of the metal ion to the CDR region of Hu1C8 played an important role in the specific recognition and binding of Hu1C8 with CD47 and the formation of the Hu1C8–CD47 complex. Indeed, an *in vitro* binding assay showed that removal of the metal ion by EDTA chelation abolished the interaction between CD47 and Hu1C8 (Fig. 5*D*). Replenishment of EDTA-treated Hu1C8 with several bivalent cations commonly found in organisms showed that compared to Fe^2+^, Mg^2+^ and Zn^2+^, Ca^2+^ could efficiently restore the binding of Hu1C8 to CD47 (Fig. 5*E*). To analyze the types and abundances of metal ions in the protein solution, we performed ICP-OES (inductively coupled plasma optical emission spectrometer) analysis and the results showed that Ca^2+^ is the most abundant (>95%) metal ion in the protein solution. As the concentration of Ca^2+^ is much higher than that of Fe^2+^, Mg^2+^, and Zn^2+^ in the cell culture medium and inside the cells, we assigned the bound metal ion in the structure as Ca^2+^ and believe that the bound Ca^2+^ is biologically relevant and plays an important role in helping Hu1C8 to create a unique antigen binding site that specifically recognizes and binds the conformational epitope of CD47.

To understand the underlying mechanism by which Hu1C8 maintains a similar activity of inhibiting cancer cell growth to Hu5F9-G4 but has a low activity to induce hemagglutination, we compared the structure of the Hu1C8-CD47 complex with that of the Hu5F9-CD47 complex (PDB code: 5IWL) (25) and the SIRPα-CD47 complex (PDB code: 2JJS) (26) (Fig. 5*F*). The structural comparison showed that although both Hu1C8 and Hu5F9 bind to the β-sheet of CD47-ECD to occupy part of the SIRPα-binding site and thus block the binding of SIRPα, they recognize and bind to different regions of the β-sheet of CD47-ECD with distinct binding orientations (Fig. 5, *F* and *G*). Specifically, SIRPα mainly binds to the upper part of the β-sheet of CD47-ECD, Hu5F9 mainly binds to the loops on the upper part of the β-sheet of CD47-ECD, and Hu1C8 mainly binds to the lower part of the β-sheet of CD47-ECD (Fig. 5, *F* and *G*). Further structural analysis of the CD47 ECD in complexes with other antibodies in the PDB databank showed that the recognition and targeting of these loops together with the upper part of the β-sheet of the CD47 ECD, seem to be a common feature of many CD47 antibodies (Fig. S5). These results indicate that Hu1C8 recognizes a unique conformational epitope on the CD47 ECD and binds to the CD47 ECD with a distinct binding orientation different from the other antibodies (Figs. 5, *F* and *G*; S5).

Further superposition of the Hu1C8-CD47 and Hu5F9-CD47 complexes onto the full-length IgG4 antibody and the full-length CD47 revealed that the different binding orientations of Hu1C8 and Hu5F9 to the CD47 ECD might yield different binding modes with CD47, leading to differences in RBC hemagglutination (Fig. 5*H*). Both Hu5F9 and Hu1C8 could bind two CD47 molecules in a Y-shaped conformation. However, as CD47 binds to one Fab of Hu5F9 in a pose almost parallel to the Fab arm, the two CD47 molecules recognized by a single full-length Hu5F9 antibody are oriented approximately perpendicular to each other’s Fab arm (Fig. 5*H* left). This binding mode provides sufficient space for the Hu5F9 antibody to bind to two CD47 molecules on different cells, leading to RBC hemagglutination. On the other hand, since CD47 binds to the Fab of Hu1C8 at an approximately 130° angle to the Fab arm, the two CD47 molecules recognized by one Hu1C8 antibody are in a nearly parallel orientation, and their spacing is narrow (Fig. 5*H* right). This binding mode is unable to allow one Hu1C8 antibody to crosslink two cells simultaneously due to its conformational changes, thus preventing RBC hemagglutination. This might explain why Hu1C8 does not tend to bind to two cells simultaneously and has low RBC toxicity.

## Discussion

Numerous studies have shown that CD47 is essential for the treatment of a variety of malignancies. Blockade of the CD47/SIRPα axis enhances macrophage-dependent phagocytosis of tumor cells, establishing this immune checkpoint as a therapeutic target in cancer immunotherapy (18). The clinical development of CD47-based therapies has achieved breakthrough progress in recent trials (27). However, this therapeutic mechanism also targets CD47 molecules on the surface of RBCs, inducing phagocytosis-mediated destruction and resulting in anemia during clinical use. Therefore, reducing RBC toxicity is a critical issue in the development of CD47 antagonists. To increase the efficacy and safety of CD47 antagonists, many next-generation anti-CD47 antibodies with reduced RBC toxicity have been developed (28, 29, 30, 31). Meanwhile, bispecific antibodies, SIRPα-Fc fusion proteins, and small-molecule inhibitors have been developed to reduce hematotoxicity. In this study, we focused on developing a novel anti-CD47 antibody with unique epitope recognition and antigen binding mode to minimize RBC toxicity.

Hemagglutination is a reaction that causes the clumping of RBCs. Specific antigens or receptors exist on the surface of RBCs. When they specifically bind to corresponding substances such as antibodies, viruses, and lectins, the crosslinking action will cause the RBCs to interconnect with each other and form agglutinated clumps. Anti-CD47 antibodies may target the CD47 on the surface of RBCs and cause cellular clumping and lattice structure formation, which can cause coagulation. Interestingly, in our structural study, when one arm of a Hu1C8 molecule bound to the unique epitope of CD47 on RBCs with distinct binding orientations, the other arm was restricted to the same cell membrane. Therefore, the complex could not act as a bridge between two cells, thereby losing the ability to induce the formation of lattice structures. The structural and mechanistic study of Hu1C8 validated the hypothesis that the Fab binding orientation of anti-CD47 antibodies correlates with hemagglutination potential (28) and provided structural insights for developing non-hemagglutinating CD47 antibodies.

In addition, these results reaffirm that epitope localization critically governs antibody activity and provides a structural basis for the rational design of therapeutic mAbs with tailored functional properties. For anti-CD47 antibodies, selecting the antigen-binding epitope is particularly important to avoid hemagglutination caused by cell crosslinking. In fact, Yu *et al.* have shown that the stimulatory activity of human anti-CD40 antibodies was shown to decreased as epitopes became closer to the cell membrane (32). They speculate that the importance of the epitope location is related to the accessibility of the Fc domain. Specifically, the Fc domain of antibodies that are closer to the membrane will not be able to optimally bind to FcγR. Anti-CD40 antibodies frequently rely on secondary crosslinking *via* Fcγ receptors (FcγR) for biological activity (33); however, excessive activation may lead to toxic side effects. In conclusion, the epitope and recognition angle of an antibody are likely to constrain the accessibility of the other arm or the Fc region, which provides valuable guidance for antibody design. By strategically leveraging or circumventing cell-crosslinking through such mechanisms, antibodies can achieve an optimal balance between therapeutic efficacy and safety.

In addition to their direct applicability in immunotherapeutics, the antibody properties elucidated in this study represent a rather rare phenomenon. The diversity of antigen-binding specificities of antibodies is generated by the genetic processes of recombination and mutation. Accumulating evidence suggests that the immune system can exploit additional strategies to diversify the repertoire of antigen specificities. These unconventional mechanisms exclusively target the antigen-binding sites of immunoglobulins and include the insertion of large amino acid sequences, posttranslational modifications, conformational heterogeneity, and use of nonprotein cofactor molecules (34). Among these, the unique ligation properties of metal ions are widely utilized by proteins, particularly the metalloproteins but are rarely exploited by antibodies. Zhou *et al.* first reported that a CD4-reactive Ab Q425 requires calcium for antigen recognition (35). In this study, we observed a similar phenomenon with a rabbit antibody against CD47. Specifically, X-ray crystallographic analyses show that Hu1C8 requires a bivalent metal ion to form robust interaction with CD47, suggesting that the immune system can exploit additional strategies to diversify the repertoire of antigen specificities.

## Experimental procedures

### Cells, antibodies, and recombinant proteins

ExpiCHO-S cells (Gibco) were cultured in ExpiCHO expression medium (Gibco) supplemented with 1% penicillin‒streptomycin (Gibco) at 37 °C in 8% CO_2_. Raji cells and CCRF-CEM cells were cultured in RPMI 1640 (Gibco) supplemented with 10% fetal bovine serum (FBS) and 2% penicillin-streptomycin at 37 °C in 5% CO_2_. HAECs and RPTECs were generated and cultured in Dulbecco’s modified Eagle’s medium (DMEM; Gibco) supplemented with 10% FBS and 2% penicillin-streptomycin at 37 °C in 5% CO_2_. These cells are from the National Collection of Authenticated Cell Cultures. Human RBCs and PBMCs were purchased from Milestone Biotechnologies.

The Hu5F9-G4 VH and Vκ sequences were commercially synthesized (Shanghai Generay Biotech Co., Ltd) in accordance with the patents (WO 2011/143,624 A2) and were cloned and inserted into the human IgG4PE scaffold. The negative control antibody (8D6) was generated in our laboratory (21). These antibodies were expressed in ExpiCHO-S cells.

### Animal immunization

The HuCD47 ECD, which had a 6 × His tag fused to the C-terminus (amino acids Gln1-Pro121; Acro Biosystems), was used as the antigen. Male New Zealand White rabbits weighing 2 to 2.5 kg were subcutaneously immunized at multiple points with 1 mg of antigen in the presence of complete Freund's adjuvant, followed by two boosters with the same dose of antigen in the presence of incomplete Freund's adjuvant. Immunization was performed every 3 weeks, and serum samples were collected 7 days after the third immunization. The rabbits were housed at the Shanghai Tengda Rabbit Industry Professional Cooperative. All animal experiments were performed in accordance with the relevant regulations of the Shanghai Tengda Rabbit Industry Professional Cooperative.

### Isolation of rabbit monoclonal antibodies

PBMCs were isolated from the blood of immunized rabbits using Lympholyte-Mammal density separation medium (Cedarlane) according to the manufacturer's instructions. The HuCD47 ECD was labelled with EZ-Link Sulfo-NHS-LC-Biotin (Thermo Fisher Scientific) as a sorting probe. PBMCs were stained with Fixable Viability Stain 510 (BD Biosciences), Alexa Fluor 647 donkey anti-rabbit IgG (BioLegend), goat anti-rabbit IgM-FITC (Southern Biotech), mouse anti-rabbit CD4-FITC (Bio-Rad), mouse anti-rabbit CD8-FITC (Bio-Rad), mouse anti-rabbit T lymphocyte-FITC (Bio-Rad), and biotinylated HuCD47 ECD-streptavidin-SA BV421. Single HuCD47 ECD-specific memory B cells (FITC^−^/APC^+^/BV421^+^) were isolated using a Sony MA900 instrument and sorted into a 96-well PCR plate containing lysis buffer. The antibody VH and Vκ in each cell were amplified by RT-PCR and nested PCR using primer panels as previously described (36). The VH and Vκ genes were sequenced, cloned and inserted into human IgG4PE and Igκ expression vectors.

### Expression and purification of antibodies

The VH and Vκ gene expression plasmids were co-transfected into ExpiCHO-S cells using an ExpiFectamine CHO Transfection Kit (Invitrogen) according to the manufacturer's instructions. The supernatants were harvested, and the rabbit monoclonal antibodies were purified using Protein A Sepharose (GE Healthcare) according to the manufacturer's instructions and dialyzed against PBS.

### ELISA

To determine the binding properties of the sera and antibodies, 96-well microwell plates (Nunc) were coated with 1 μg/ml HuCD47 ECD or CyCD47 ECD (Acro Biosystems) protein in 0.1 M PBS and incubated overnight at 4 °C, followed by blocking with 2% bovine serum albumin (BSA) in PBST (0.05% Tween-20 in PBS) for 2 h. The plates were washed and incubated with serially diluted sera or antibodies at 37 °C for 2 h. The samples were washed three times, and depending on the sample, horseradish peroxidase (HRP)-conjugated goat anti-rabbit IgG antibody (1:4000; R&D Systems), or anti-human Fc HRP antibody (Sigma-Aldrich, cat. no. A0170) was used to detect the bound antibodies. The EC_50_ was calculated using Prism 8.

To test the ability of the antibodies to block the binding of CD47 to its receptor SIRPα, SIRPα-Fc (Acro Biosystems) was precoated on 96-well plates, and serially diluted antibodies were incubated with the HuCD47 ECD for 1 h at 37 °C. The mixture was subsequently added to the plates and incubated at 37 °C. After 1 h, the plates were washed, and the HuCD47 ECD that bound to SIRPα was examined with an HRP-conjugated mouse anti-His monoclonal antibody. The 50% inhibitory concentration (IC_50_) was calculated using Prism 8.

### FACS-based binding and blocking assays

To assess whether the antibodies could bind to CD47 on the surface of cells, serially diluted antibodies were incubated with tumor cells, normal cells, or RBCs that had been stained with Fixable Viability Stain 780 (BD Biosciences) at 4 °C in the dark for 30 min. The cells were subsequently washed three times, and the antibodies bound to the cells were examined with FITC-conjugated anti-human IgG (BioLegend), followed by FACS analysis with a BD Fortessa. For blocking assays, serially diluted antibodies were incubated with Fixable Viability Stain 780-stained tumor cells in the presence of 5 μg/ml bio-SIRPα-Fc (SIRPα-Fc labelled with EZ-Link Sulfo-NHS-LC-Biotin) at 4 °C for 30 min. After the samples were washed, the bound bio-SIRPα-Fc was examined using streptavidin-conjugated BV421 (BD Biosciences), followed by FACS analysis with a BD Fortessa. The data were analyzed by FlowJo V10 and Prism 8.

### Biolayer interferometry (BLI) analysis

Biolayer interferometry was performed using an Octet Red96 instrument (ForteBio, Inc.) to analyze the *K*_d_ between the antibodies and CD47. 5 μg/ml antibody solution was immobilized on an anti-human IgG-Fc-coated biosensor surface for 360 s. The baseline interference phase was measured for 180 s in kinetics buffer (KB: 1 × PBS, 0.1% BSA, and 0.02% Tween-20). The sensors were then immersed in 2-fold serial dilutions of HuCD47 ECD or CyCD47 ECD in KB to examine the association phase for 360 s, followed by immersion of the sensors in KB for 600 s for the dissociation phase. The mean *K*_on_, *K*_off_, and apparent *K*_d_ values were determined from all binding curves that were globally fit to a 1:1 Langmuir binding model with an R^2^ value ≥ 0.95 by ForteBio Data Analysis 7.0 software.

### Hemagglutination analysis

To evaluate the hemagglutination capacity of the antibodies, human RBCs were washed and diluted 2% in PBS in a V-bottom-shaped 96-well plate. Then, the antibodies were serially diluted 5-fold from 100 μg/ml and added to the RBCs at a 1:1 volume ratio. The plates were incubated at room temperature for 1 to 2 h. Evidence of hemagglutination is the presence of unsettled RBCs, which appears as a diffuse haze compared to the punctate red dot of nonhemagglutinized RBCs.

### *In vitro* phagocytosis assay

Human PBMC suspensions (6 × 10^5^ cells/ml) in RPMI 1640 containing 50 ng/ml M-CSF (Sino Biological) were plated in 96-well plates (PerkinElmer) at 100 μl/well and differentiated into macrophages for 6 to 7 days until the monocyte-derived macrophages (MDMs) became adherent and the other cells were washed away by culture media. CCRF-CEM cells were labelled with CellTrace Blue (Invitrogen) at 37 °C in the dark for 20 min. The CCRF-CEM cells were then washed and resuspended at 6 × 10^5^ cells/ml in RPMI 1640 medium, followed by being added to MDMs at 50 μl/well. Meanwhile, different concentrations of antibodies were added. Phagocytosis was allowed for 3 h at 37 °C in the dark. Since CCRF-CEM cells are suspension cells, the non-phagocytosed CCRF-CEM cells were removed by washing with culture media three times. The MDMs were stained with FITC-conjugated anti-human CD14 (BioLegend) *in situ*. Phagocytosis was analyzed using an Opera (PerkinElmer). Macrophages were identified by green fluorescence (488 nm) and enumerated. Phagocytosed tumor cells were quantified *via* blue fluorescence (365 nm). The number of phagocytosis-positive macrophages was recorded using green fluorescence that encapsulates the blue fluorescence. The phagocytic ratio was calculated as the proportion of tumor cell-engulfing macrophages relative to the total macrophage population.

### Antitumor activity of the antibodies *in vivo*

M-NSG mice xenografted with Raji tumor cells were used to evaluate the antitumor activity of Hu1C8. When the tumor volume reached approximately 80 mm^3^, the mice were randomized into seven groups and then treated with the antibodies. The antibodies were injected intraperitoneally once every 3 days for six continuous doses. Body weight and the width and length of the tumors were measured every 3 days, and the following formula was used: tumor volume = length x width x width/2. On Day 18, the mice were sacrificed, and the tumor tissues were collected for both photographic documentation and weighing. The mice were housed under pathogen-free conditions. This protocol for animal experiments was approved by the Institutional Animal Care and Use Committee of the Center for Excellence in Molecular and Cellular Sciences.

### Antibody humanization

The rabbit antibodies were humanized by a CDR-grafting strategy. CDRs in the heavy and light chains of antibodies were identified by a combination of a 3D structure model and the Abysis online tools (http://www.abysis.org/abysis/). The online platform allows users to input antibody sequences and extract CDR information according to the database you have selected, such as Kabat and IMGT. The sequences of the rabbit antibodies were aligned with the human germline VH and Vκ databases to identify the most closely related human germline sequences for CDR grafting. After the CDRs were grafted, human back to rabbit mutations were introduced in the key residues involved in the function of the antibody in the FRs. The sequences of humanization antibodies were commercially synthesized and cloned into human IgG4PE and Igκ expression vectors. The expression and purification of the humanized antibodies were performed as previously described.

### Crystallization, data collection, and structure determination

The Fab fragment of Hu1C8 was generated by papain digestion of the Hu1C8 antibody and purified by Protein A agarose (GE Healthcare) and gel filtration on a Superdex 200 10/300 column (Cytiva) in buffer containing 20 mM Tris pH 7.5 and 150 mM NaCl.The CD47 ECD (residues 1–121), with a C15A mutation (26) and C-terminal 6 histidine tag, was cloned into pcDNA3.4. CD47 ECD was expressed transiently in ExpiCHO-S cells in the presence of 8.6 μM Kifunensine. CD47 ECD were purified by HisTrap excel resin (Cytiva) according to the manufacturer's instructions. To further reduce the oligosaccharide chains attached on protein surface, CD47 ECD was incubated with endoglycosidase-H (endoH) at 37 °C for 1 h followed by size exclusion chromatography purification with a Superdex 75 column (GE Healthcare) and the fraction containing polished CD47 ECD was collected. The purified Fab Hu1C8 was mixed with CD47 ECD at 1:1 stoichiometric ratio and the protein complex was then concentrated to 15 mg/ml prior to crystallization experiments.

Crystallization was carried out at 16 °C using the hanging-drop vapor diffusion method by mixing equal volumes (1 μl) of the Hu1C8 Fab-CD47 ECD complex solution and the reservoir solution. Crystals of the Hu1C8 Fab-CD47 ECD complex were grown in drops containing a reservoir solution of 0.1 M HEPES (pH 7.0) and 20% (w/v) polyethylene glycol 3350. The crystals were transferred into the cryoprotectant consisting of the reservoir solution and 20% (v/v) glycerol for cryoprotection, followed by flash-cooling into liquid nitrogen. X-ray diffraction data were collected at BL18U1 of National Facility for Protein Science Shanghai. Diffraction data indexing, integration, and scaling were performed using HKL2000 (37). The structure of the Hu1C8 Fab-CD47 ECD complex was solved by molecular replacement method as implemented in Phenix (38) using the crystal structures of anti-HSA Fab (PDB code 5FUZ) (39) and CD47 ECD (PDB code 2JJS) (26) as the search models. Model building was performed using Coot (40), and structure refinement was performed using Phenix (38). The stereochemistry and quality of the structure model were analyzed using programs in the CCP4 suite (41). The statistics of the diffraction data, the structure refinement and the final structure model are summarized in Table S1.

## Data availability

The crystal structure of human CD47 ECD bound to Fab of Hu1C8 has been deposited in the Protein Data Bank with accession code 8ZCA (https://www.rcsb.org/structure/8ZCA). All other data are available in the main text or in the supporting information section.

## Supporting information

This article contains supporting information (25, 26, 42, 43, 44).

## Conflict of interest

The authors declare that they have no conflicts of interest with the contents of this article.
