# Supplementary material for: An anti-CD47 antibody binds to a distinct epitope in a novel metal ion-dependent manner to minimize cross-linking of red blood cells
Source: J Biol Chem. 2025 Jun 25;301(8):110420. doi: 10.1016/j.jbc.2025.110420 (PMC12302720; doi:10.1016/j.jbc.2025.110420)
Supplement: Supporting information [file mmc1.docx]

**An anti-CD47 antibody binds to a distinct epitope in a novel metal ion-dependent manner to minimize cross-linking of red blood cells**

Xiao Lu^1#^, Ziyue Chen^2#^, Chunyan Yi^1#^, Zhiyang Ling^1#^, Jing Ye^4#^, Kaijian Chen^2^, Yao Cong^2^, Sonam Wangmo^4^, Shipeng Cheng^1^, Ran Wang^5^, Danyan Zhang^4^, Jiefang Xu^5^, Jichao Yang^4^, Liyan Ma^1^, Qing Duan^6^, Xiaoyu Sun^3*^, Jianping Ding^2,4*^ and Bing Sun^1, 4*^

^1^Key Laboratory of Multi-Cell Systems, Shanghai Institute of Biochemistry and Cell Biology, Center for Excellence in Molecular Cell Science, University of Chinese Academy of Sciences, Chinese Academy of Sciences, Shanghai 200031, China.

^2^Key Laboratory of RNA Innovation, Science and Engineering, Shanghai Institute of Biochemistry and Cell Biology, Center for Excellence in Molecular Cell Science, University of Chinese Academy of Sciences, Chinese Academy of Sciences, Shanghai 200031, China.

^3^Shanghai Institute of Infectious Disease and Biosecurity, Shanghai Medical College, Fudan University, Shanghai 200032, China.

^4^School of Life Science and Technology, ShanghaiTech University, Shanghai 201210, China.

^5^Division of Life Sciences and Medicine, University of Science and Technology of China, Hefei, China.

^6^TOT BIOPHARM Company Limited, Jiangsu 215024, China.

^#^ Xiao Lu, Ziyue Chen, Chunyan Yi, Zhiyang Ling and Jing Ye contributed equally to this work.

* Corresponding authors:

Bing Sun: [bsun@sibs.ac.cn](mailto:bsun@sibs.ac.cn); Jianping Ding: [jpding@sibcb.ac.cn](mailto:jpding@sibcb.ac.cn); Xiaoyu Sun: [sunxiaoyu@fudan.edu.cn](mailto:sunxiaoyu@fudan.edu.cn);


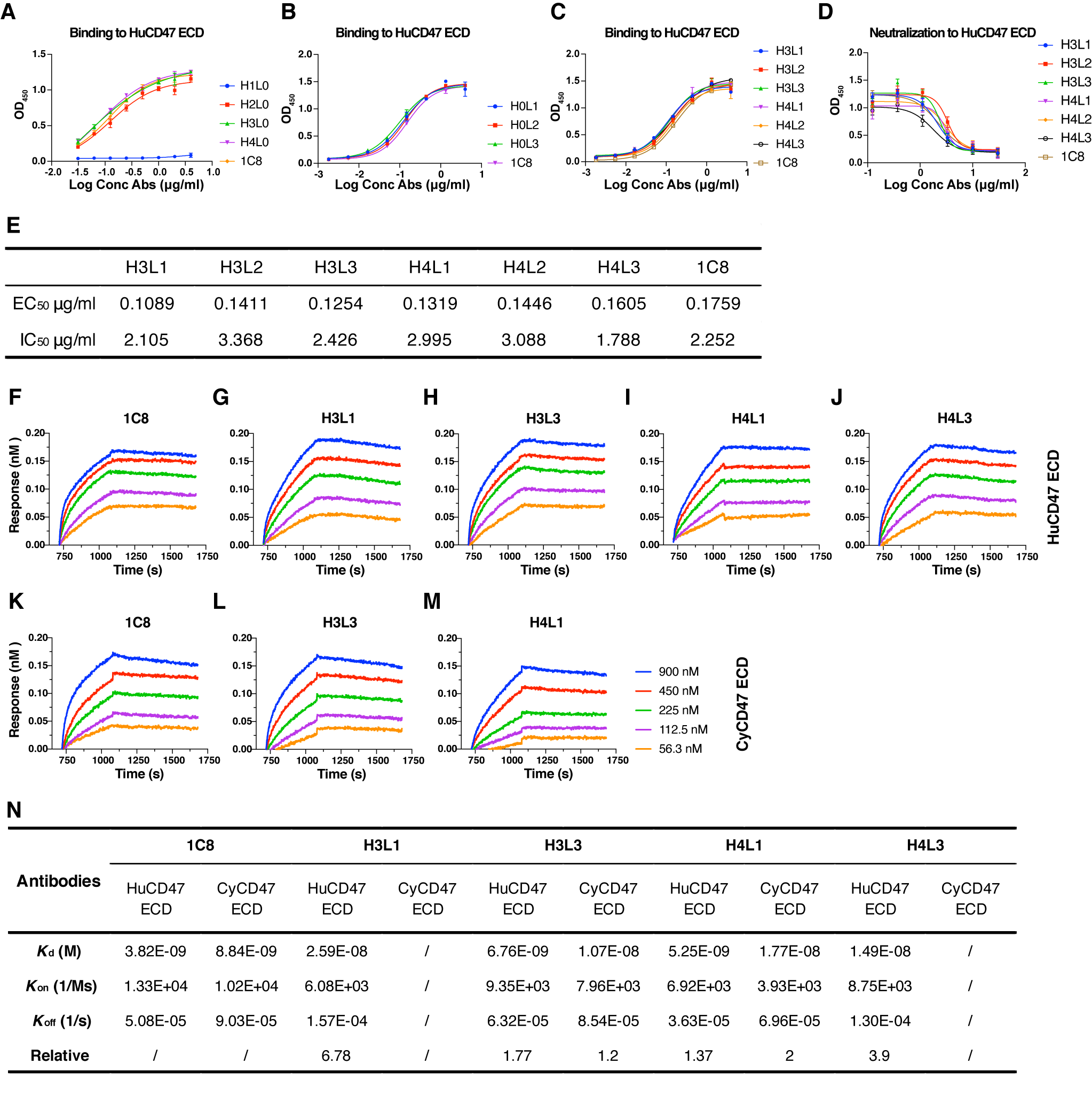


**Figure S1 The activity of humanized antibodies. A-B** Binding activity of the first round of humanized antibodies to the HuCD47 ECD. **C** Binding activity of the second round of humanized antibodies to the HuCD47 ECD. **D** Neutralizing activity of the second round of humanized antibodies against the HuCD47 ECD. **E** The EC_50_ and IC_50_ values of the humanized antibodies. **F-M** Fitting curves of the binding kinetics of humanized antibodies to the HuCD47 ECD (F-J) and the CyCD47 ECD (K-M), which were evaluated by the “1:1 Langmuir binding model” with an R2 value ≥0.95 by Fortebio Data Analysis 7.0 software. **N** Affinities (*K*_d_ values) of the humanized antibodies for the HuCD47 ECD and the CyCD47 ECD. The results in (**A-D**) are presented as the means ±SD. Data are representative of two independent experiments.


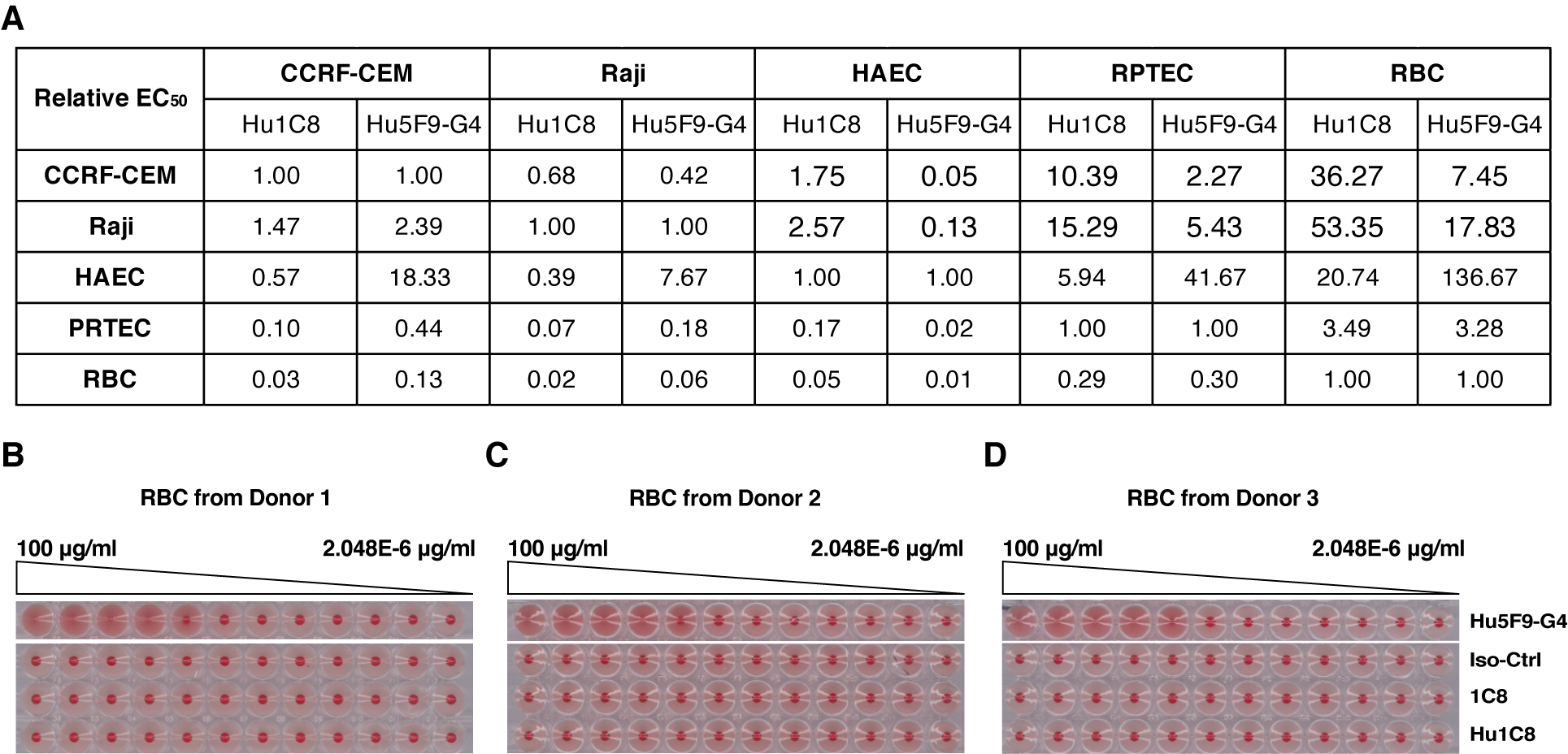


**Figure S2 The binding activity of Hu1C8 to different cells. A** The relative binding activity of Hu1C8 to different normal and tumour cells. **B-D** Haemagglutination activity of Hu1C8 on human RBCs from different donors.


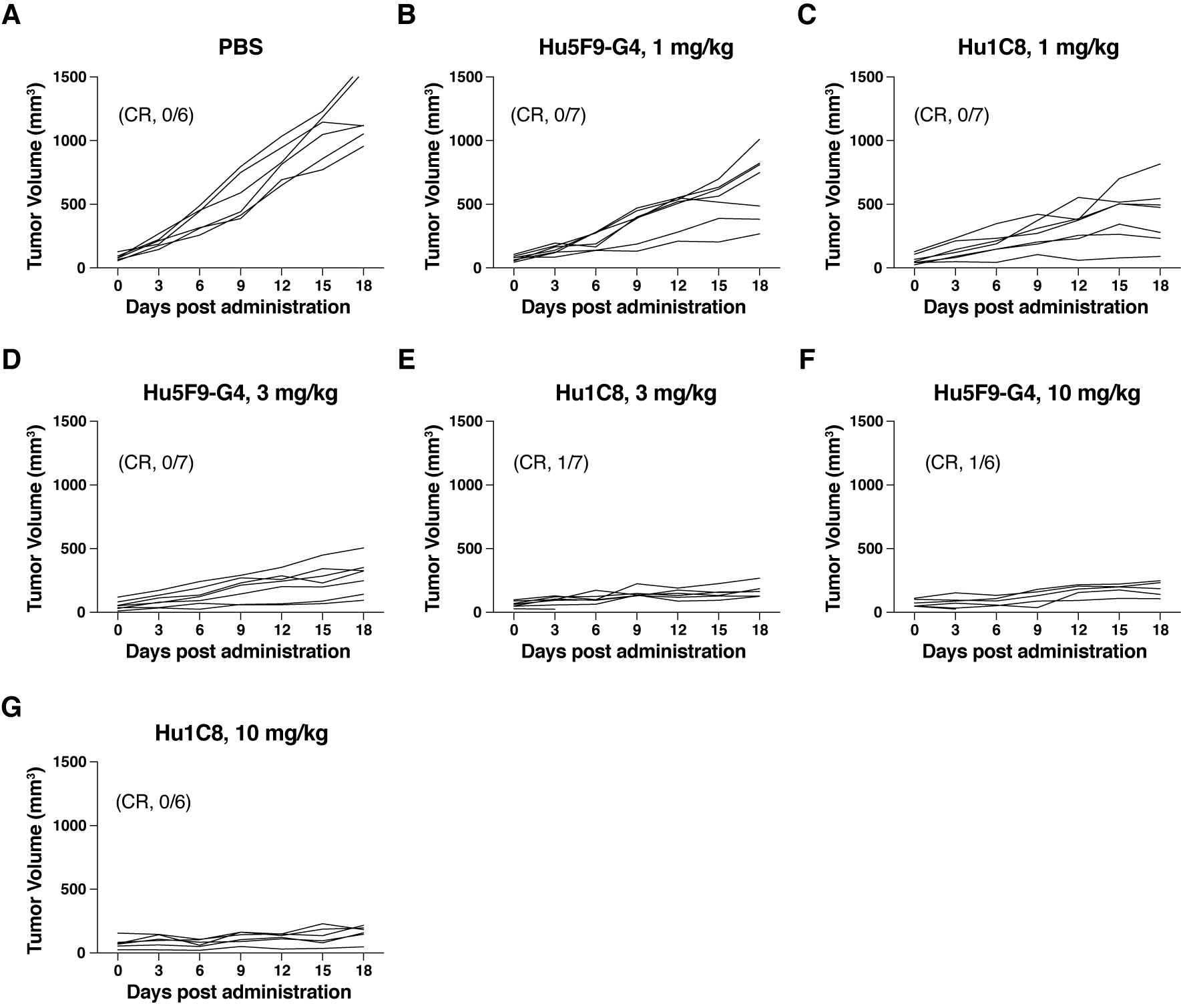


**Figure S3 Hu1C8 inhibited tumour growth in a dose-dependent manner *in vivo*. A-C** The curves of tumor volume nder different treatment regimens (n=6-7 mice per group). CR, Complete Response.


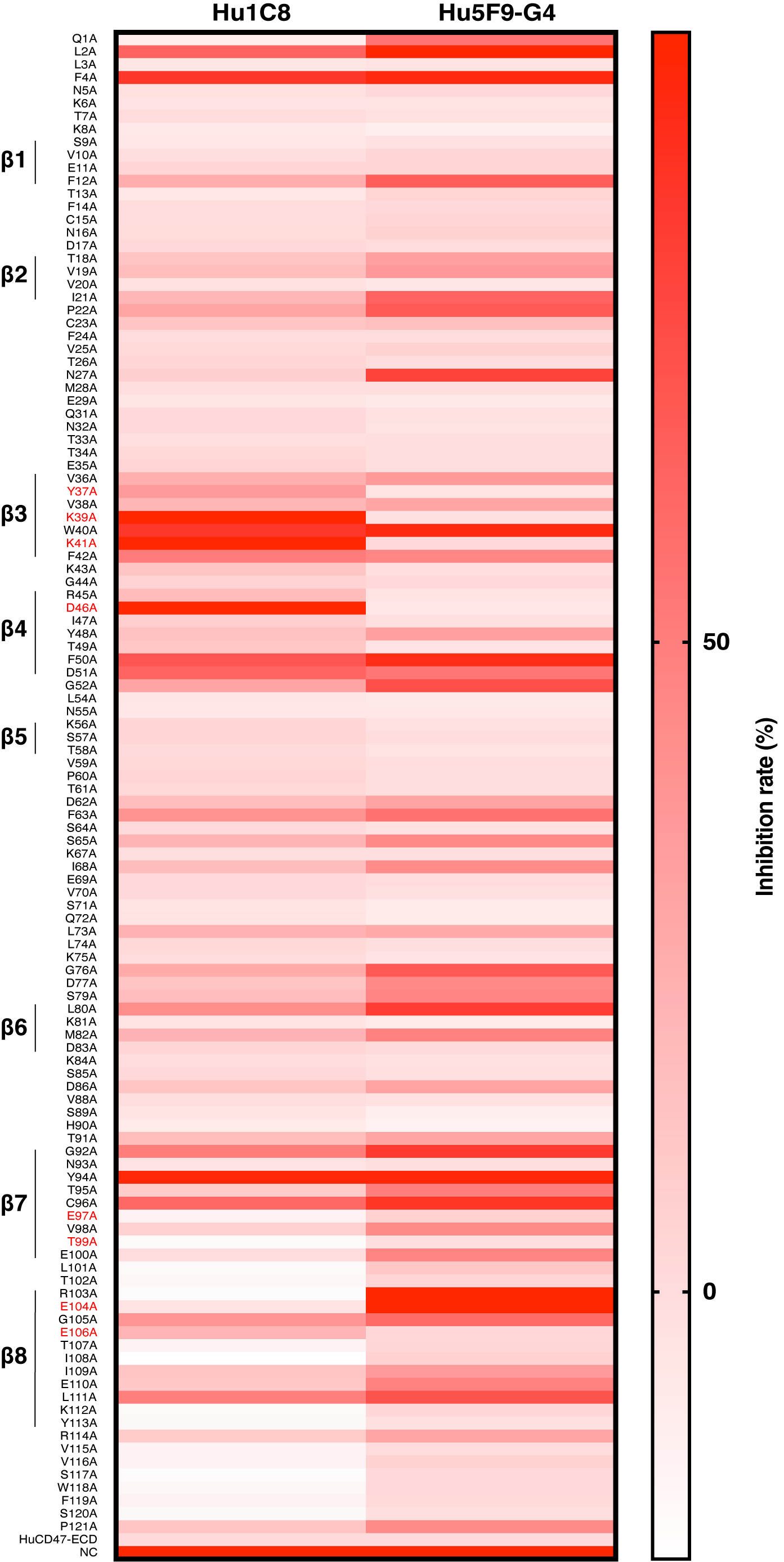


**Figure S4 Effect of different alanine variants of the HuCD47 ECD on the inhibition rate of antibody binding.** The inhibition rate was calculated as the HuCD47 ECD value minus the mutant well value divided by the HuCD47 ECD value.


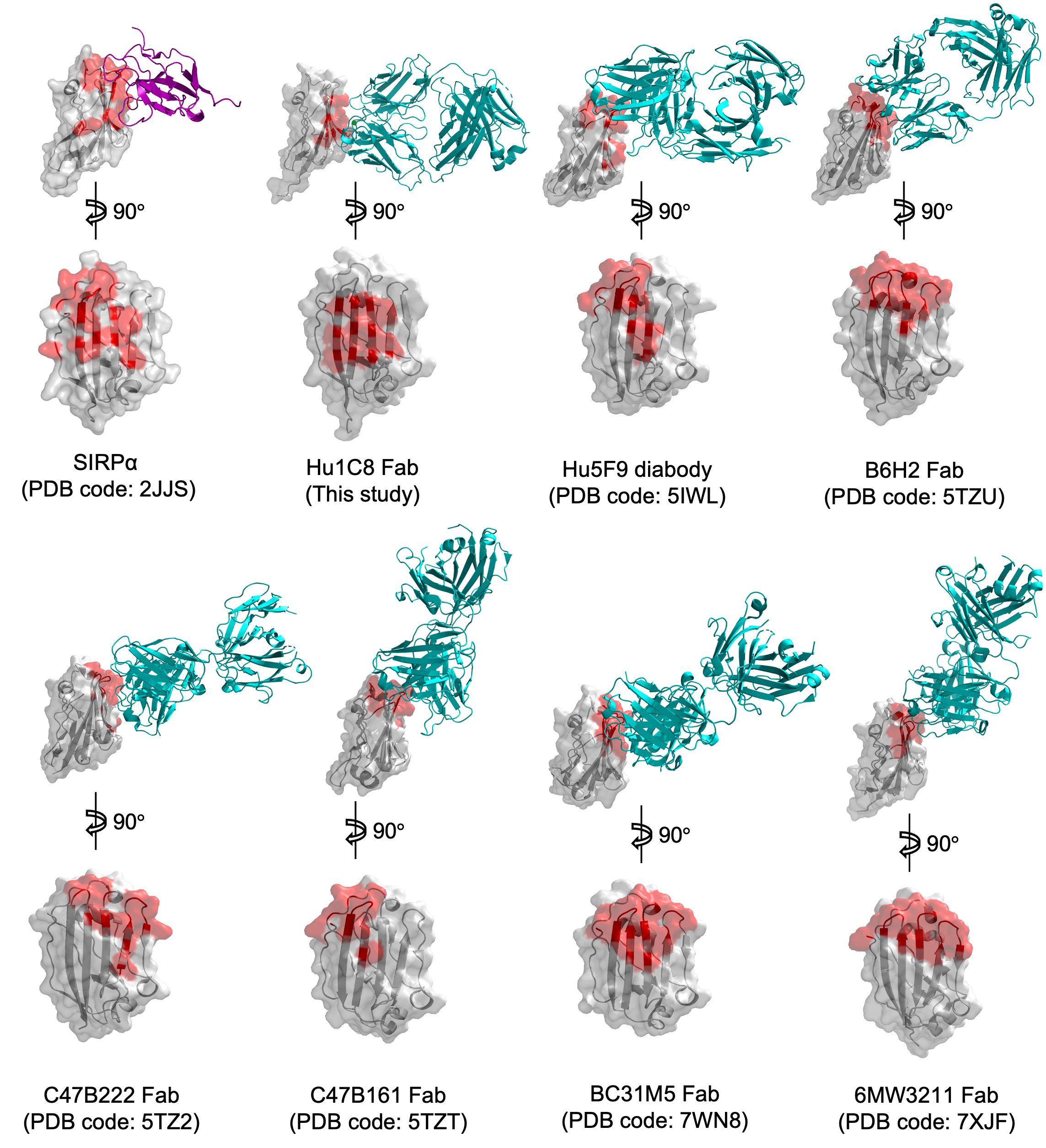


**Figure S5 Structural comparison of the CD47-SIRPα^1^, CD47-Hu1C8 and other CD47-antibody complexes****^2, 3, 4, 5^.** The CD47 ECD, SIRPα and the Fab or diabody of different antibodies are shown in gray, purple and cyan, respectively. The epitope residues on the CD47 ECD are highlighted in red. The upper panel and the lower panel show two different orientations of the CD47 ECD.

**Supplementary Table 1. Crystallographic diffraction data and structure refinement statistics**

|  | **Hu1C8 Fab-CD47 ECD** |
| --- | --- |
| **Data collection** |  |
| Wavelength (Å) | 0.9792 |
| Resolution (Å) | 50.0-2.49 (2.58-2.49) ^a^ |
| Space group | *P* 1 |
| Cell parameters  *a*, *b*, *c* (Å) | 45.22, 79.93, 92.75 |
| *α*, *β*, *γ* (°) | 112.51, 96.61, 103.16 |
| Observed reflections | 108,105 |
| Unique reflections (I/σ(I) > 0) | 38,120 (2572) |
| Average redundancy | 2.8 (2.4) |
| Average *I*/σ(*I*) | 8.1 (2.8) |
| Completeness (%) | 99.6 (99.2) |
| R_merge_ (%) ^b^ | 16.3 (33.5) |
| CC_1/2_ | 0.955 (0.858) |
| **Refinement and structure model** |  |
| Reflections (*Fo≥0σ(Fo*)) | 36,286 (2571) |
| Working set | 34,384 (2436) |
| Test set | 1902 (135) |
| R_work_ / R_free_ (%) ^c^ | 21.5/26.6 |
| No. of atoms | 8565 |
| Protein | 8323 |
| Ligands | 70 |
| Solvent | 172 |
| Wilson B-factor (Å^2^) | 41.02 |
| Average B-factor (Å^2^) | 45.50 |
| Protein | 45.53 |
| Ligands | 44.86 |
| Solvent | 44.31 |
| RMS deviations |  |
| Bond length (Å) | 0.009 |
| Bond angles (°) | 1.06 |
| Ramachandran plot (%) |  |
| Favoured | 95.22 |
| Allowed | 4.78 |
| Outliers | 0 |

^a^ Numbers in parentheses refer to the highest resolution shell.

^b^ R_merge_=∑_hkl_∑_i_⎪*I*_i_(*hkl*)_i_−〈*I*(*hkl*)〉⎪/∑_hkl_∑_i_*I*_i_(*hkl*).

^c^ R factor=⎪⎪*F_o_*⎪-⎪*F_c_*⎪⎪/⎪*F_o_*⎪.

**References**

1. Hatherley D, Graham SC, Turner J, Harlos K, Stuart DI, Barclay AN. Paired receptor specificity explained by structures of signal regulatory proteins alone and complexed with CD47. *Mol Cell* **31**, 266-277 (2008).

2. Li Y*, et al.* A pH-dependent anti-CD47 antibody that selectively targets solid tumors and improves therapeutic efficacy and safety. *J Hematol Oncol* **16**, 2 (2023).

3. Pietsch EC*, et al.* Anti-leukemic activity and tolerability of anti-human CD47 monoclonal antibodies. *Blood Cancer J* **7**, e536 (2017).

4. Wang R*, et al.* Blockade of dual immune checkpoint inhibitory signals with a CD47/PD-L1 bispecific antibody for cancer treatment. *Theranostics* **13**, 148-160 (2023).

5. Weiskopf K*, et al.* CD47-blocking immunotherapies stimulate macrophage-mediated destruction of small-cell lung cancer. *J Clin Invest* **126**, 2610-2620 (2016).
